# Elasticity of the Cervix in Relation to Uterus Position

**DOI:** 10.3390/jcm13092572

**Published:** 2024-04-27

**Authors:** Anjeza Xholli, Ambrogio Pietro Londero, Umberto Scovazzi, Angelo Cagnacci

**Affiliations:** 1 Teaching Unit of Obstetrics and Gynecology, IRCCS Ospedale San Martino of Genova, 16132 Genova, Italy; anj160583@yahoo.it (A.X.); umbe8.rhcp@hotmail.it (U.S.); 2 Department of Neurology, Rehabilitation, Ophthalmology, Genetics, Maternal and Infant Health (DiNOGMI), University of Genova, 16132 Genoa, Italy; ambrogiopietro.londero@unige.it

**Keywords:** retroversion, retroflexion, elastography, cervix, stiffness, cervix structure, uterine flexion

## Abstract

Strain elastography allows the evaluation of tissue elasticity. **Background/Objectives:** Tissue elasticity depends on the content and distribution of collagen fibers and is shaped by the applied tensile forces that may differ in uteri with a different angle of flexion of the corpus on the cervix. The objective was to investigate whether the angle of uterine flexion is related to cervical tissue elasticity. **Methods:** The anterior angle between the longitudinal axis of the uterus corpus and that of the cervix was measured in 275 non-pregnant young women by transvaginal ultrasonography and considered both as an absolute value or categorized as ≤150°, between >150° and ≤210°, and >210°. Strain elastography was used to assess tissue elasticity by placing the probe in the anterior vaginal fornix. Tissue elasticity was evaluated in the middle of the anterior cervical compartment (ACC), in the middle of the posterior cervical compartment (PCC), in the middle portion of the cervical canal (MCC), and at the internal cervical os (ICO). In a sagittal plane MCC was evaluated across the cervical canal, and ACC and PCC at a distance equal between the cervical canal and the outer anterior or posterior part of the cervix. MCC, ACC and PCC were evaluated at equal distance between the ICO and the external cervical os. Elasticity was expressed as a color score ranging from 0.1 (low elasticity) to 3 (high elasticity). **Results:** The angle of uterine flexion show a negative linear relation with the elasticity of the ACC (*p* = 0.001) and MCC (*p* = 0.002) and a positive relation with the elasticity of the PCC (*p* = 0.054). In comparison to uteri with an angle of flexion of <150°, those with an angle of flexion of >210° had lower elasticity of the ACC (*p* = 0.001) and MCC (*p* = 0.001) and higher elasticity of the PCC (*p* = 0.004). The ACC/PCC and PCC/MCC elasticity ratios were also significantly different (*p* = 0.001). **Conclusions:** The angle of uterine flexion is associated with changes in cervix elasticity. Retroflexion is associated with stiffer ACC and MCC and a more elastic PCC. Differences in tissue elasticity suggest structural changes of the cervix that may have implication in variate obstetric and gynecological conditions.

## 1. Introduction

The uterine cervix comprises collagen fibers, ground matrix, and a substantial amount of muscle at the internal os, which gradually diminishes towards the external os [1,2,3]. According to biochemical data, imaging techniques, and mathematical models, the quantity and direction of collagen fibers change throughout the cervix [4,5], most likely due to the action of various tensile and dilatative forces [6,7]. Fiber distribution characterizes the capability of the cervix to resist to both dilatative and compressive forces [6]. During pregnancy, specific areas, such as the internal cervical os and possibly the upper posterior cervix, receive [8,9] and counteract [5,9] the fetus’s tensile and dilatative forces. Collagen fibers play an essential role in maintaining cervix competence, and when they decrease and disperse, cervix dilation may occur [2,5,10]. Changes in these processes can result in preterm birth or prolonged labor [11,12]. Because of differences in tensile forces exerted by the uterosacral and cardinal ligaments [6,7,11] or by the uterus corpus [13], the direction and quantity of collagen fibers of the cervix may differ in uteri with different positions. Along with ground matrix, the amount and direction of collagen fibers, determine tissue elasticity [2,10,14]. Strain elastography (SE) is an ultrasonographic technique for determining tissue elasticity [12,15,16,17,18], and it has been widely used to assess the elastography characteristics of cervix in both pregnant [16,19,20,21,22] and non-pregnant [23,24] women. In this study we evaluated whether the elastography characteristics of the cervix change with different uterine positions. Ligaments that maintain the uterus within the pelvis are essentially the uterosacral ligaments, and, to less extent, the cardinal ligaments that produce an elastic bounding of the cervix within the pelvis, while the round ligaments create an elastic bounding aimed to maintain the uterus corpus in its position. There are physiological variants of uterine positions, that can be described by uterine version and uterine flexion. Version is defined by the relation between the longitudinal axis of the uterus and that of the vagina, while flexion is defined by the anterior angle between the longitudinal axis of the uterine corpus and the longitudinal axis of the cervix [25,26]. In general, anteversion and retroversion indicate a uterus that forms an anterior angle with the vagina 180° < or > to 180°, respectively. The angle of flexion is in general expressed as a continuous value, and in clinical studies it was also categorized as an angle < 150°, to identify strong anteflexion, between 150 and 210° to identify no marked flexion, and >210°, to indicate a strong retroflexion [25,26]. The different angles of flexion were associated with a different intensity of menstrual pain [25], and a different risk of adenomyosis [26]. Determinants of uterine position are still elusive, but the tensile forces exerted by external ligaments and by the uterine corpus in uteri of different position can possibly determine a different structure of the cervix. This study aimed to evaluate whether the angle of uterine flexion is related to changes in the elastography characteristics of the cervix.

## 2. Materials and Methods

The local ethical committee approved this observational study (CER Liguria 123/2022) that was performed on 275 premenopausal women of the outpatient services of infertility and chronic pelvic pain at a university hospital. Each woman signed an informed consent form authorizing the anonymous use of her clinical data in scientific publications, and she was managed per standard clinical practice. Data were collected in an electronic database and then anonymously retrieved and analyzed.

**Figure 1:**
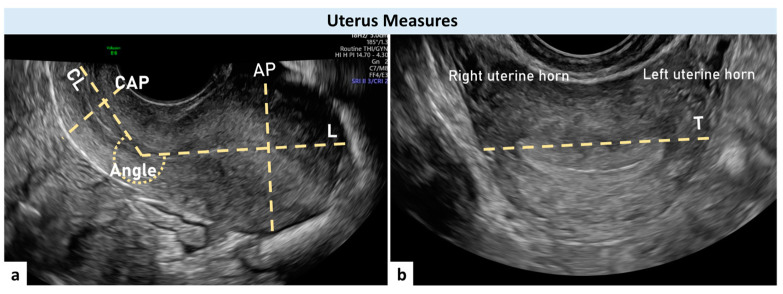
Ultrasonographic image of a retroflexed uterus in a sagittal (**a**) and transverse scan (**b**). The interrupted lines indicate uterus measures. L: longitudinal uterus measure; AP: anterior-posterior uterus measure; CAP: cervix antero-posterior diameter; CL: cervix length; ICO: internal cervical os; ECO: external cervical os; Angle: angle of uterine flexion; Probe: ultrasound probe; T: transverse uterus measure.

For each woman, we collected general characteristics and clinical data. The presence of gynecological diseases was evaluated by patient history, bimanual examination, and transvaginal ultrasonography. The ultrasound investigation was performed by a single experienced sonographer (A.X.) using a GE E6 (GE Medical Systems, Zipf, Austria) ultrasound machine equipped with a wideband 5–9 MHz intravaginal transducer and proper software for elastography (Voluson E6 BT16, GE Medical System, Zipf, Austria). Longitudinal (L), transverse (T), and antero-posterior (AP) measures of the uterus, length (CL) and diameter (CD) of the cervix, and degrees of the angle of flexion of the uterus on the cervix were obtained during the ultrasound examination. The anterior angle between the cervix’s longitudinal axis and the uterine body’s longitudinal axis was measured with an empty bladder (Figure 1). Three measures were recorded for each patient, and the mean of the three was used in subsequent analyses. The volume of the uterus was calculated by the ultrasound machine based on the formula (L × T × AP × 0.5223) and that of the cervix by the cylinder formula (CL × [CT/2] × 3.14). The uterine L and AP measures were taken in a sagittal plane, with the entire endometrial cavity visible. L was measured between the internal cervical os (ICO) and the more prominent aspect of the fundus; AP measure was estimated perpendicular to L, with the calipers placed at the most prominent parts of the uterus corpus from the anterior to posterior wall serosa. T was measured in a transverse plane (by rotating the vaginal probe 90° and placing the calipers from serosa to serosa at the Fallopian tube insertion) (Figure 1).

**Figure 2:**
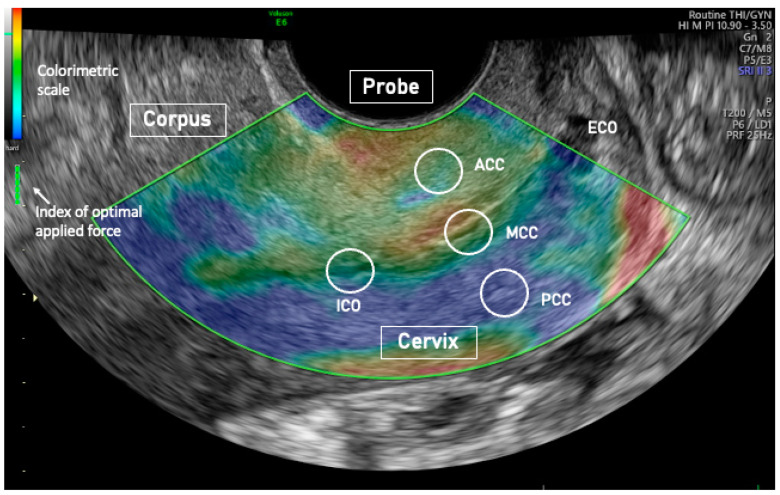
Elastography image of the cervix of an anteflexed uterus showing a stiffer posterior than anterior (cervical compartment stiffer (blue) than an anterior (cyan/green) cervical compartment. Probe: transvaginal elastography probe; ICO: internal cervical os; ACC: anterior cervical compartment; MCC: middle cervical canal; PCC: posterior cervical compartment; ECO: external cervical os.

The longitudinal cervix diameter was calculated as a line drawn between the ICO and the external cervical os (ECO). The cervix antero-posterior and transverse diameters were calculated as two orthogonal lines drawn at the mid-cervix on their respective planes. For statistical purposes, the mean of the two was used as the cervix diameter (CD) (Figure 1). The ICO was defined as the point at which the endometrium disappears, and the cervical canal begins. SE analysis was performed in a sagittal plane view with the probe directed almost perpendicular to the cervical canal (Figure 2) [16,19,24].

SE was used to evaluate tissue elasticity. SE evaluates differences in elasticity of different regions of interest (ROIs) [13,14] by measuring tissue deformation or displacement generated by the applied pressure. The procedure was already described elsewhere [24]. During image acquisition, vaginal probe was positioned in the anterior vaginal fornix and displayed alongside to facilitate images interpretation [26]. A series of about 5 compression and decompression cycles, using sub-centimetric excursions, were applied perpendicular to the axis of the cervical canal [16,19,24] (Figure 2). Optimal compression force was identified in real time by a control bar of the ultrasound processing program. Analyses were recorded on clips and analyzed afterward. ROIs with a circular area of 19.6 mm2 were placed in the middle of the anterior cervical compartment (ACC), in the middle of the posterior cervical compartment (PCC), in the middle portion of the cervical canal (MCC), and at the ICO of the cervix. In a sagittal plane, ROIs were placed at equal distances from the internal and external cervical os (Figure 2). The MCC ROI was placed across the cervical canal, and the ACC and PCC were seated at a distance equal between the cervical canal and the outer anterior or posterior part of the cervix. Strain results were calculated at optimal compression force defined by the elastography software (General electrics Company, Boston, MA, USA) (Figure 2). Three independent scorers coded tissue elasticity using a colorimetric scale ranging from violet/blue (low elasticity) to red (high elasticity), with yellow/green serving as intermediate values. The scorers assigned values to each ROI analysis across the entire colorimetric spectrum from 0.1 = blue/violet to 3.0 = red. SE analysis inter-operator ICC agreement was 0.93 (95% CI 0.89,0.96). The average of the 3 scorers’ scores was used. We used univariate and multiple linear regression analyses to examine the relationship between the angle of flexion of the uterus on the cervix (dependent variable) and independent variables such as woman age, age at menarche, body mass index (BMI), number of at-term pregnancies and deliveries, uterus and cervix measurements, ROIs elasticity, and the calculated ratio of ROIs elasticities. Only the independent variables that in simple regression analysis were related to the dependent variable (up to a *p* value of 0.2) were entered into the multiple regression models. In the multiple regression models the variables that were not significantly associated with the dependent variable were gradually eliminated, beginning with the least relevant, to finally preserve only those variables that were independently related to the dependent variable. As previously performed in studies on menstrual pain [25] and adenomyosis [26], the angle of flexion of the uterus on the cervix was also categorized as ≤150°, between >150° and ≤210°, and >210°.

To compare means from different groups, one-way ANOVA (analysis of variance) was used, followed by Fisher’s Least Significant Difference post hoc test. Statview 5.1 was used to conduct statistical analyses (SAS Institute Inc., Cary, NC, USA). The normality of continuous variables was tested with the Kolmogorow-Smirnoff test. The information is presented as a mean and standard deviation (SD), and a *p*-value < 0.05 was considered statistically significant.

## 3. Results

Clinical characteristics and measures of the uterus, of the included women, are reported in Table 1.

### 3.1. Elasticity of the Cervix

Cervical tissue was not homogeneous, and elasticity progressively increased from the ICO, the PCC, the ACC, and the MCC (Table 1). The angle of flexion was positively related to PCC elasticity (*p* = 0.001), to PCC/MCC elasticity ratio (*p* = 0.001), and negatively, to ACC elasticity (*p* = 0.001) and ACC/PCC elasticity ratio (*p* = 0.001) (Table 2). In addition, the angle of uterine flexion was related to age (*p* = 0.036), uterus volume (*p* = 0.009), AP (*p* = 0.001), T (*p* = 0.018), and the ratio AP/cervix diameter (*p* = 0.001) (Table 2).

### 3.2. Multiple Regression Models

Three models were calculated by multiple regression analyses (Table 3).

In Model 1, we entered only the variables significantly related to the angle of uterine flexion. In this calculation (R^2^ = 0.116), an independent negative relation with the angle of uterine flexion were found for ACC elasticity (*p* = 0.001), and cervix diameter (*p* = 0.037) and a positive relation with AP measure of the uterus (*p* = 0.001). In model 2 measures of the uterus and cervix were included as single variable represented by the ratio of the uterus AP measure/cervix diameter. In this model (R^2^ = 0.124), the angle of flexion was negatively related to the color score of the ACC (*p* = 0.001), and positively related to the ratio of the uterus AP measure/cervix diameter (*p* = 0.001). In model 3, we included the ratio of elasticity of different ROIs. The model achieved a slightly higher relation between dependent and independent variables (R^2^ = 0.163). The angle of uterine flexion remained positively related to the uterus AP measure/cervix diameter (*p* = 0.001), and the elasticity ratio of the PCC/MCC (*p* = 0.001).

### 3.3. Data Stratification by Angle of Uterine Flexion

**Figure 3:**
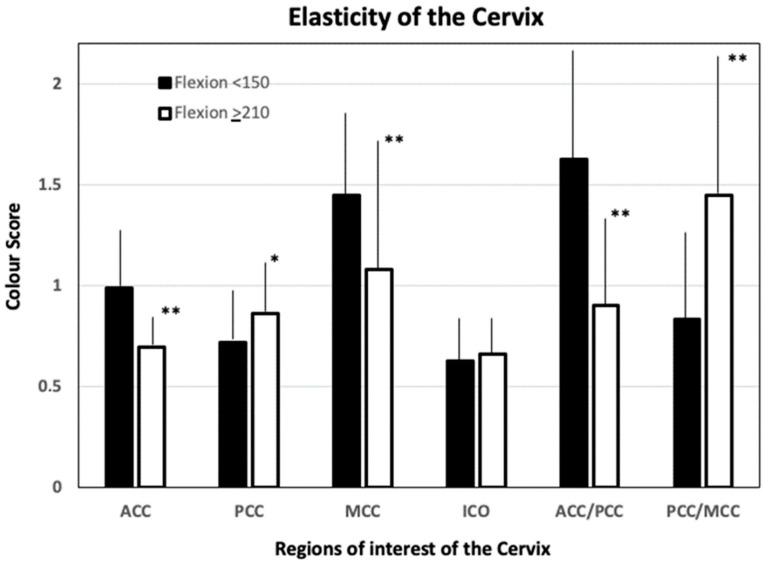
Mean (SD) values of tissue elasticity of the anterior cervical compartment (ACC), posterior cervical compartment (PCC), middle cervical canal compartment (MCC), internal cervical os (ICO), and their elasticity ratio, observed in uteri with an angle of flexion ≤150° or >210° * *p* = 0.004; ** *p* = 0.001.

**Figure 4:**
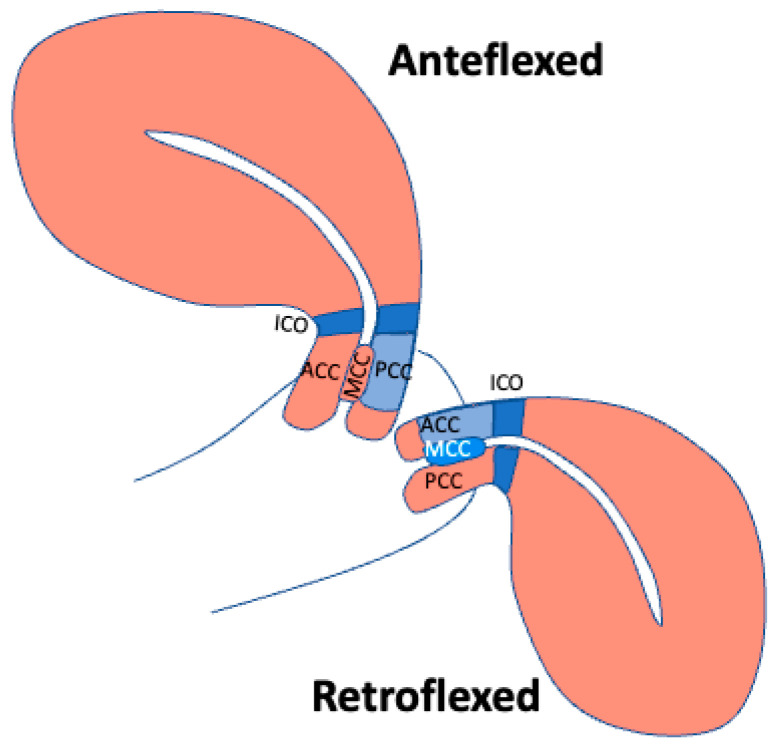
Schematic representation of the elasticity of different cervical areas observed in anteflexed (left) and retroflexed (right) uteri. ACC: anterior cervical compartment; PCC: posterior cervical compartment; MCC: middle cervical canal compartment; ICO: internal cervical os. Elasticity increases with the intensity from blue up to pink.

Among included women 213 (77.4%) had an angle of flexions ≤150°, 21 (7.6%) women an angle of flexion between >150° and ≤210°, and 41 (14.9%) an angle of flexion >210°. Parameters that were significantly different among these groups are reported in Table 4. In comparison to uteri with an angle of flexion ≤150°, those with an angle of flexion >210° had a significantly higher PCC elasticity (*p* = 0.004) and lower diameter of the cervix (*p* = 0.009). Conversely, ACC (*p* = 0.001) and MCC (*p* = 0.001) elasticities were lower. The elasticity ratio of the ACC/PCC and the PCC/MCC were also significantly different between uteri with an angle of flexion >210° and ≤150° (*p* = 0.001) (Figure 3 and Figure 4).

## 4. Discussion

### 4.1. Principal Findings

The angle of uterine flexion seems to be related to elasticity changes in some areas of the cervix like the ACC, the PCC, and the MCC. Data stratification clearly shows that in comparison to an anteflexed, a retroflexed uterus has a stiffer ACC and MCC and a softer PCC (Figure 4).

### 4.2. Results in the Context of What Is Known

Ground matrix, collagen fibers, and their grade of anisotropy determines tissue stiffness [2,10,14]. It is unclear which component is mostly modified in relation to the angle of uterine flexion, but the data strongly suggest that the ACC, the PCC, and the MCC structures are different in uteri with different angles of flexion.

### 4.3. Clinical Implications

It is unknown whether the difference in cervix elasticity is primary or secondary to the angle of flexion. In the first case, it indicates that women may have a primitive difference in the structure of their cervix that influences the angle of flexion of their uterus. Thus, changing this angle artificially through surgery [27,28,29] or possibly during pregnancy may not affect the structure of the cervix. In contrast, whether the cervix structure is secondary to the angle of flexion, i.e., shaped temporarily by differences in external or internal tensile forces, artificial modification of the angle may result in cervical structural changes. A different structure of the cervix may have implications in pregnancy, anteflexed uteri possibly responding differently to dilatative forces than retroflexed uteri. This possibility would deserve dedicated studies to be investigated.

### 4.4. Research Implications

Published investigations on cervix structure based on histology, imaging techniques, and mathematical models never considered the position of the uterus as a confounding factor [1,2,3,4,5,6,7,8,9,10]. Future studies need to incorporate the angle of flexion of the uterus among the variables that may influence the results. The possible implications of a different composition of the cervix linked to the angle of flexion should be considered in association with different obstetrics and gynecological disturbances and pathologies. It was already reported that a higher variance of ROIs elasticity of the cervix or a different angle of uterine flexion is associated with menstrual pain [24,25] risk of adenomyosis [26,30] and infertility [23]. Furthermore, some evidence indicates that during pregnancy, the angle of flexion may be related to preterm birth [31].

### 4.5. Strengths and Limitations

The study was conducted on women recruited from outpatient infertility and chronic pelvic pain services at a university hospital. Other researchers must replicate the findings in different settings and populations. A limit of the study is that we have no data on eventual surgeries, performed on the cervix. Surgery may affect cervix elasticity, but there is no reason to suspect a different rate of surgeries in uteri with different uterine flexion. SE is a semiquantitative analysis, and values of tissue elasticity can differ depending on the applied force, on the operator that performs the analysis, and on the interpretation of the color score results. We attempted to overcome some of these limitations by executing the analysis at the optimal compression force suggested by the elastography software and by expressing the data as the ratio between two ROIs that were investigated simultaneously during the same force externally applied by the investigator [18,24]. A single operator performed all ultrasound and elastography evaluations, and three researchers scored the colors of the elastography analysis. Results of tissue elasticity by SE can be influenced by the distance of the tissue from the transducer or by the interposition of the cervical canal, the distant cervical lips receiving a reduced applied force. The consequent reduced tissue compression/decompression may give the false impression of a higher stiffness. In our analysis, as also previously reported [26], the probe was always placed in the anterior cervical fornix thus reducing the possibility that the distance of the anterior and posterior lips differs among ante-flexed and retro-flexed uteri. In linear regression models, tissue elasticity was related to the angle of uterine flexion with the angle of flexion between >150° and ≤210°, showing values intermediate between the marked ante-flexion (≤150°) and the marked retro-flexion (>210°), as to indicate a progression of elasticity changes. Furthermore, the MCC is equidistant from the transducer, independently whether the probe is applied to the anterior or posterior cervical lip, and its elasticity differs among ante- and retro-flexed uteri. Thus, overall, the data indicate that the angle of uterine flexion, more than confounding, is related to the elasticity of cervical tissues evaluated by SE.

## 5. Conclusions

The current data show that the signal obtained by SE of different areas of the cervix varies with the angle of uterine flexion. These differences may indicate structural differences of the cervix that may have clinical implications in obstetric and gynecological conditions.

## Figures and Tables

**Table 1:** Demographic information, uterine measurements, and elasticity of four cervical regions of interest, measured by strain elastography in 275 women.

Parameter	Value
Age (yrs.)	36.7 ± 7.5
Menarche (yrs.)	12.6 ± 1.6
BMI (Kg/m^2^)	22.9 ± 4.8
Abortion (%)	4.1
Parous (%)	20.5
Caesarean delivery (%)	4.5
Uterus Volume (mm^3^)	61.9 ± 45.3
Uterus L (mm)	59.5 ± 13.6
Uterus AP (mm)	39.0 ± 10.4
Uterus T (mm)	50.9 ± 10.5
Cervix volume (mm^3^)	20.1 ± 6.18
Cervix length (mm)	26.6 ± 5.19
Cervix diameter (mm)	23.5 ± 4.9
ACC elasticity	0.94 ± 0.36
PCC elasticity	0.74 ± 0.29
MCC elasticity	1.39 ± 0.51
ICO elasticity	0.63 ± 0.28

Data are expressed as mean (SD) or percentage. L: longitudinal measure; AP: antero-posterior measure; T: transverse measure; ACC: anterior cervical compartment; PCC: posterior cervical compartment; MCC: middle cervical canal compartment; ICO Internal cervical os.

**Table 2:** Simple linear regression analyses between the angle of uterine of flexion (dependent variable) and significantly related parameters, in 275 women.

Parameter	R^2^	Coefficient of Regression	95% Confidence Interval	*p* Value
Age (yrs.)	0.013	0.828	0.054; 1.601	0.036
Uterus Volume (mm^3^)	0.021	0.173	0.042; 0.303	0.009
Uterus AP (mm)	0.040	0.970	0.421; 1.520	0.001
Uterus T (mm)	0.017	0.670	0.116; 1.225	0.018
Cervix Diameter (mm)	0.010	−1.175	−2.376; 0.002	0.059
Uterus AP/Cervix diameter	0.065	23.67	13.09; 34.28	0.001
ACC elasticity	0.071	−36.2	−51.51; −20.913	0.002
PCC elasticity	0.010	19.6	−0.360; 39.710	0.054
MCC elasticity	0.047	−26.611	−32.81; −10.40	0.002
ACC/PCC	0.490	−11.34	−17.68; −5.60	0.001
PCC/MCC	0.109	28.44	18.87; 38.02	0.001

Data are expressed as mean (SD) or percentage. AP: anterior-posterior measure; T: transverse measure; ACC: anterior cervical compartment; PCC: posterior cervical compartment; MCC: middle cervical canal compartment.

**Table 3:** Multiple linear regression models between the angle of uterine flexion and the independently related factors evaluated in 275 women.

Parameter	Coefficient of Regression	95% Confidence Interval	*p* Value
Model 1 (R^2^ = 0.116)			
Uterus AP (mm)	1.014	0.461; 1.567	0.001
Cervix Diameter (mm)	−1.24	−2.401; −0.075	0.037
ACC color score	−33.9	−49.2; −18.65	0.001
Model 2 (R^2^ = 0.124)			
Uterus AP/Cervix diameter	21.66	11.34; 31.89	0.001
ACC color score	−33.4	−48.6; −18.2	0.001
Model 3 (R^2^ = 0.163)			
Uterus AP/Cervix diameter	21.67	11.60; 31.75	0.001
PCC/MCC	26.83	17.41; 32.21	0.001

Data are expressed as mean (SD) or percentage. AP: anterior-posterior measure; ACC: anterior cervical compartment; PCC: posterior cervical compartment; MCC: middle cervical canal compartment.

**Table 4:** Demographic, ultrasonographic characteristics and elastography parameters of the cervix of 275 women categorized accordingly to the angle of uterine flexion (A: ≤150°; B: >150°–≤210°; and C: >210°).

Parameter	A	B	C	*p* A vs. B	*p* A vs. C	*p* B vs. C
Age (yrs.)	35.3 ± 7.1	41.8 ± 7.1	34.7 ± 8.2	0.001	0.632	0.004
Menarche (Yrs.)	12.5 ± 1.7	12.8 ± 1.5	12.7 ± 1.2	0.723	0.718	0.907
BMI (Kg/m^2^)	22.7 ± 4.8	24.6 ± 5.2	23.1 ± 4.7	0.131	0.692	0.288
Uterus Volume (mm^3^)	56.7 ± 35.40	86.1 ± 86.9	65.9 ± 51.1	0.004	0.232	0.089
Uterus L (mm)	54.0 ± 11.4	56.8 ± 21.6	53.7 ± 14.80	0.356	0.890	0.383
Uterus AP (mm)	37.7 ± 8.8	48.4 ± 16.1	40.4 ± 11.8	0.001	0.132	0.004
Uterus T (mm)	50.2 ± 9.7	56.8 ± 16.6	51.3 ± 10.1	0.007	0.520	0.060
Cervix volume (mm^3^)	19.8 ± 5.8	22.0 ± 8.5	19.9 ± 6.0	0.115	0.090	0.016
Cervix Diameter (mm)	23.9 ± 4.6	23.4 ± 6.5	21.6 ± 5.2	0.693	0.009	0.182
ACC elasticity	0.98 ± 0.37	0.89 ± 0.3	0.69 ± 0.26	0.239	0.001	0.044
PCC elasticity	0.72 ± 0.290	0.71 ± 0.25	0.86 ± 0.24	0.859	0.004	0.046
MCC elasticity	1.44 ± 0.51	1.44 ± 0.59	1.08 ± 0.36	0.976	0.001	0.010
ICO elasticity	0.62 ± 0.28	0.58 ± 0.29	0.66 ± 0.23	0.442	0.425	0.246
ACC/PCC	1.62 ± 1.03	1.39 ± 0.77	0.90 ± 0.53	0.292	0.001	0.058
PCC/MCC	0.83 ± 0.49	0.89 ± 0.41	1.45 ± 0.76	0.612	0.001	0.001

L: longitudinal measure; AP: anterior-posterior measure; T: transverse measure; ACC: anterior cervical compartment; PCC: posterior cervical compartment; MCC: middle cervical canal compartment; ICO: internal cervical orifice.

## Data Availability

Due to ethics restriction the data are available from the authors.
